# Effects of Sodium Acetate Supplementation on Growth, Hematologic and Plasma Biochemical Parameter, Lipid Deposition, and Intestinal Health of Juvenile Golden Pompano *Trachinotus ovatus* Fed High-Lipid Diets

**DOI:** 10.1155/2024/7904141

**Published:** 2024-09-07

**Authors:** Pengwei Xun, Siling Zhuang, Handong Yao, Jinhao Su, Yukai Yang, Hu Shu, Wei Yu, Heizhao Lin

**Affiliations:** ^1^ School of Fisheries Xinyang Agriculture and Forestry University, Xinyang 464000, China; ^2^ School of Life Science Guangzhou University, Guangzhou 510006, China; ^3^ Key Laboratory of South China Sea Fishery Resources Exploitation and Utilization Ministry of Agriculture and Rural Affairs South China Sea Fisheries Research Institute Chinese Academy of Fishery Sciences, Guangzhou 510300, China; ^4^ Shenzhen Base of South China Sea Fisheries Research Institute Chinese Academy of Fishery Sciences, Shenzhen 518121, China; ^5^ Tropical Fisheries Research and Development Center South China Sea Fisheries Research Institute Chinese Academy of Fishery Sciences, Sanya 572018, China

## Abstract

Experimental diets were formulated including the suitable lipid level (10%, PC), the high-lipid level (16%, HL), and HL containing sodium acetate diets (HS). Three diets were fed golden pompano (*Trachinotus ovatus*) (initial body weight: 12.88 ± 0.03 g) for 8 weeks. The results showed HL diets significantly increased hepatosomatic index (HSI) and abdominal fat percentage (ASF), aggravated liver lipid deposition, and caused blood metabolic disorder and liver damage (*P* < 0.05). Moreover, the fish fed HL diets significantly decreased intestinal villus number (VN) and muscular layer thickness (MLT) (*P* < 0.05), accompanied with an increased trend in the relative abundance of intestinal pathogenic bacteria such as *Mycoplasma* and *Photobacterium*. However, the fish fed HS diets significantly decreased the HSI and AFP, relieved hepatic lipid deposition, improved blood and liver metabolism, and intestinal morphology in comparison to the fish fed HL diets (*P* < 0.05). More importantly, sodium acetate addition improved intestinal microbiota by inhibiting the proportion of pathogens (*Mycoplasma* and *Vibrio*) and increasing the abundance of probiotics (*Bacteroidales_S24-7_group_norank*, *Cetobacterium*, *Bacteroides*, and *Lachnospiraceae_NK4A136_group*). Furthermore, there was a strong correlation between these bacteria (*Mycoplasma*, *Vibrio*, *Lachnospiraceae_NK4A136_group*, *Bacteroidales_S24-7_group_norank*, *Bacteroides*, and *Cetobacterium*) and main physiological indices. In conclusion, sodium acetate improved blood performance, alleviated hepatic lipid deposition induced by HL diets, and boosted the growth and intestinal health for golden pompano.

## 1. Introduction

Golden pompano (*Trachinotus ovatus*) is a carnivorous marine fish, which has important economic values because of its tasty flavour and various nutrition [1]. Given predacity of golden pompano, it necessitates a substantial amount of protein in its diet, especially fishmeal. In recent years, the inadequate supply of fishmeal and the escalating demand from aquatic feed production has resulted in the substantial rise in fishmeal prices [2]. High-lipid diet (HL) has been widely used in the cultivation of golden pompano considering its protein-sparing and growth-promoting functions [3]. However, the adverse effects induced by HL like poor growth, liver lipid deposition, disorder of blood biochemical indicators, and intestinal inflammation [4, 5, 6, 7, 8] have contributed to gigantic economic loss to the industry of golden pompano.

Recent studies have highlighted the potential of dietary additives in reducing the risk of fatty liver and keeping the homeostasis of intestinal microbiota in fish. Short-chain fatty acids (SCFAs) have been confirmed numerous biological functions, including adjusting metabolism, reducing oxidative stress, boosting immunity, and fostering growth [9]. Acetate, as a major SCFAs, has been demonstrated to modulate lipid oxidation and *de novo* synthesis through the AMPK pathway [10]. Moreover, acetic acid can reduce lipid synthesis and storage by inhibiting insulin signaling in adipose tissue [11]. Sodium acetate is one of acetate, which can be hydrolyzed into CH_3_COO^−^ and Na^+^. Acetic acid also exists in the form of free CH_3_COO^−^ in the organism. So the action mechanism of sodium acetate and acetic acid in the organism is the same. Considering the stability and convenience, sodium acetate is usually selected as feed additive to explore the effect of acetic acid on aquatic animals. In aquatic livestock, the roles of acetic acid in relieving HL-induced detrimental impacts have been reported in several studies. For example, sodium acetate mitigated lipid accumulations induced by sodium palmitate in blunt snout bream (*Megalobrama amblycephala*) [12]. Inclusions of sodium acetate relieved HL-caused lipid deposit in liver and improved liver health status in Nile tilapia (*Oreochromis niloticus*) [13]. A recent study showed sodium acetate promoted growth, ameliorated hepatic lipid deposition, and enhanced effects for oxidation resistance of *Onychostoma macrolepis* fed HL diets [14]. However, the role played by sodium acetate in *T. ovatus* fed HL diets has not been reported.

Gut microbial community is essential for maintaining the health of the host. Many evidences have suggested that feeding HL diets is one of the important factors leading to intestinal imbalance [15, 16, 17, 18], and the dysbiosis of gut microorganism would cause metabolic disturbance [19]. Improvement of metabolic diseases by regulation of intestinal microbiota have attracted increasing interest of nutritionist [20]. In human and animal models, it has been confirmed strong connections between HL diet, obesity, and intestinal microbiota [21, 22]. Sodium acetate has also been confirmed to boost carbohydrate utilization of largemouth bass (*Micropterus salmoides*) by regulating intestinal microorganism [23]. However, existing researches about the effects of sodium acetate on fish fed HL diets mainly focused on the lipid deposition and health status of liver. Whether the sodium acetate can alleviate the lipidosis and tissue damage caused by HL diets by regulating intestinal microorganisms remains unknown.

Thus, the aim of this study was to estimate the influences of sodium acetate on growth, major physiological parameters, and gut microbiota of *T. ovatus* fed HL diets and to explore the correlation between gut microbiota and major physiological parameters.

## 2. Materials and Methods

### 2.1. Experimental Diets

The experimental formula is presented in Table 1. Our previous research has determined that the optimal sodium acetate supplementation level for *T. ovatus* is 0.14%, where the growth and physiological state of the fish are the best [24]. Therefore, three experimental diets were formulated including the suitable lipid level (10%, PC), the high-lipid level (16%, HL), and HL supplemented with 0.14% sodium acetate (HS). The production of feed was conducted based on our prior research [17]. The prepared diets were air-dried and deposited in −20°C till use.

### 2.2. Fish Husbandry

Juvenile golden pompano was procured from Shenzhen Longqizhuang Aquatic Products Company (Shenzhen, China). Before the formal trial, the fish need to go through a 14-day acclimation period in the net cage of pond. Then, 180 juvenile fish (initial body weight: 12.88 ± 0.03 g) were randomly divided into nine net cages (1.8 m^3^, 20 fish each cage) in a pond, and each diet was distributed to three cages. During the 8-week culture, the juveniles were fed twice daily (7 : 00 and 17 : 00) for satiety, and the water-quality indices were as follows: temperature 31.06 ± 0.30°C, salinity 15.32 ± 0.20 ‰, pH 8.24 ± 0.20, dissolved oxygen content 7.75 ± 0.05 mg/L, dissolved ammonia ≤0.05 mg/L, and nitrite ≤0.01 mg/L.

### 2.3. Sample Collection

Fish was fasted 24 hr before sampling and then anaesthetized using eugenol at a concentration of 100 mg/L (Shanghai Medical Instruments). Total quantity and weight of fish in each cage were detected for calculating survival rate, final body weight (FBW), weight gain rate (WGR), specific growth rate (SGR), and feed conversion ratio (FCR). Then five fishes from each cage were measured the weight and length for calculating the condition factor (CF). Subsequently, the blood of these five fishes was sampled via caudal venipuncture using 2.5 ml heparin-treated syringes. A 0.5 ml blood sample was promptly moved to anticoagulant tubes for assessment of hematological indices. The remaining blood samples were subjected to centrifugation to extract plasma at 3,000 rpm for 15 min at 4°C and preserved at −80°C until use. Next, the viscus and liver of the fish were taken out aseptically and weighed to calculate abdominal fat percentage (AFP), viscerosomatic index (VSI), and hepatosomatic index (HSI). Finally, the liver was collected and split into two sections. One part was soaked in 4% paraformaldehyde for the detection of the histology, and the other was preserved at −80°C for measurement of lipid metabolism indices. The dorsal muscle was sampled for proximate composition and texture characteristics analysis. The intestinal content of the five fishes was removed for the assessment of gut microbiome. The midgut samples were obtained and soaked in 4% paraformaldehyde for the test of the histology.

### 2.4. Calculation

The indices were determined by the following formulae:

(1) $$\begin{array}{c} \text{WGR}\left(\%\right)=100\%\times \left(\text{Final body weight }\left(g\right)-\text{initial body weight }\left(g\right)\right)/\left(\text{initial body weight }\left(g\right)\right), \end{array}$$

(2) $$\begin{array}{c} \begin{array}{c} \text{SGR}\left(\%/d\right)=100\%\\ \times \left(\text{Ln}\left(\text{final body weight}\right)-\text{Ln}\left(\text{initial body weight}\right)\right)/\\ \left(\text{the duration of the experiment }\left(\text{days}\right)\right), \end{array} \end{array}$$

(3) $$\begin{array}{c} \text{FCR}=\left(\text{Feed intake }\left(g\right)\right)/\left(\text{weight gain }\left(g\right)\right), \end{array}$$

(4) $$\begin{array}{c} \text{Survival rate}\left(\%\right)=100\%\times \left(\text{Final number of fish}\right)/\left(\text{initial number of fish}\right), \end{array}$$

(5) $$\begin{array}{c} \text{AFP}\left(\%\right)=100\%\times \left(\text{Abdominal fat weight }\left(g\right)\right)/\left(\text{whole body weight }\left(g\right)\right), \end{array}$$

(6) $$\begin{array}{c} \text{CF }\left(g/{\left(\text{cm}\right)}^{3}\right)=100\%\times \left(\text{Body weight }\left(g\right)\right)/{\left(\text{body length}\left(\text{cm}\right)\right)}^{3}, \end{array}$$

(7) $$\begin{array}{c} \text{HSI}\left(\%\right)=100\%\times \left(\text{Liver weight }\left(g\right)\right)/\left(\text{whole body weight }\left(g\right)\right), \end{array}$$

(8) $$\begin{array}{c} \text{VSI}\left(\%\right)=100\%\times \left(\text{Viscera weight }\left(g\right)\right)/\left(\text{whole body weight }\left(g\right)\right). \end{array}$$

### 2.5. Proximate Composition

The composition of muscle was determined referring to the method [25]. Moisture contents were determined in the oven (105°C) until constant weight. Crude protein and crude lipid contents were obtained by Kjeldahl nitrogen and Soxhlet extraction, respectively. The samples were burned in a muffle furnace (550°C) for the determination of the ash contents.

### 2.6. Texture Characteristics of Dorsal Muscle

The muscle samples were sectioned into 10 mm cylindrical profiles and then determined in a texture analyzer (TC3, Brookfield, USA) referring to the method [26]. The analyzer was configured with the following settings: TA44 spherical probe, 3 mm displacement, test speed of 0.1 cm/s, and trigger threshold of 5G unit.

### 2.7. Determination of Hematologic and Plasma Biochemical Parameters

The concentration of total protein (TP), triglyceride (TG), total cholesterol (TC), glucose (GLU), alanine aminotransferase (ALT), aspartate aminotransferase (AST), and glutathione reductase (GR) in plasma were analyzed by automated biochemical analyzer. The blood samples were detected for the levels of white blood cell (WBC), red blood cell (RBC), lymphocyte (Lym), hemoglobin (HGB), hematocrit (HCT), mean corpuscular volume (MCV), mean corpuscular hemoglobin (MCH), and mean corpuscular hemoglobin concentration (MCHC) using a blood cell analyzer (Mindray, BC-5000 Vet). The blood performance (BP) was determined according to the formulae [27].

(9) $$\begin{array}{c} \text{Blood performance}=\text{ln WBC}+\text{ln RBC}+\text{ln HGB}+\text{ln HCT}+\text{ln TP}. \end{array}$$

### 2.8. Hepatic Lipid Metabolism Parameters Determination

The liver samples were homogenized with in the prechilled homogenization liquid. The supernatant was collected postcentrifugation of homogenates (3,000 rpm, 4°C) for 20 min. The hepatic triglyceride (HTG), hormone-sensitive lipase (HSL), and fatty acid synthetase (FAS) contents were tested by the absorbance microplate reader using the corresponding kits (Nanjing, China).

### 2.9. Histology Determination

The histomorphology detection of liver and midgut samples was implemented as described previously [28]. First, liver and intestine samples underwent ethanol dehydration and paraffin embedding, and were cut into sections (4 *μ*m). Then liver sections were subjected to the oil red O staining, and the relative area stained with oil red O solution was quantified using Image-Pro Plus 6.0 software. Intestinal sections were colored with H&E and photographed, and the morphological parameters were determined by case analysis software 3DHISTECH (V.2.4, Budapest, Hungary).

### 2.10. Intestinal Microbiota

The microbial 16S rRNA analysis was determined referring to the previous method [17]. First, total DNA of microbiota was extracted using the E.Z.N.A.® Stool DNA kit (D4015, Omega, Inc.). The V3 to V4 regions of 16S rRNA genes were subjected to amplification using the forward primer 341F (5′-CCTACGGGNGGCWGCAG-3′) and the reverse primer 805R (5′-GACTACHVGGGTATctaATCC-3′). Then PCR amplification was performed, and the PCR-amplified DNA products were separated by electrophoresis, purified, and quantified. The sequences were further processed on the Illumina MiSeq PE300 sequencing platform. Finally, bioinformatics test of the sequence data was performed using the QIIME (V1.9.1) for evaluating the characteristic of intestinal microbiota.

### 2.11. Statistical Analysis

All outcomes were expressed as mean ± SEM (standard error of the mean). The normality and homoscedasticity of the data were verified and then were estimated through one-way ANOVA, and Duncan's test was employed to detect the significance among the groups using SPSS21.0. The figures on physiological indexes were drawn with GraphPad Prism 8.0. The differences with *P* < 0.05 was statistically significant. Spearman analysis between intestinal microbiota and main physiological indices was conducted using the R stats package (V3.6.3). Mantel test was performed with ggplot2 package (V3.3.3), linkET package (V0.0.7.4), and psych package (V2.4.1) under R software (V4.1.3). Correlation network heatmap with signs were drawn using the OmicStudio tools at https://www.omicstudio.cn.

## 3. Results

### 3.1. Growth Performance and Body Morphologic Indices

The results of growth performance are presented in Table 2. Compared with the PC group, FBW, WGR, and SGR in the HS group were significantly raised and FCR was markedly reduced (*P* < 0.05), whereas these parameters in the HL group had no significant difference (*P* > 0.05). The body morphologic indices in Table 3 showed the fish in the HL group had obviously higher HSI and AFP than that in the PC group (*P* < 0.05), while sodium acetate supplementation in HL diet reduced these phenomena (*P* < 0.05). The fish fed HS diets had a significantly higher CF than that fed the other two diets (*P* < 0.05). Statistical difference in survival rate and VSI was not found among the three groups (*P* > 0.05).

### 3.2. Proximate Composition and Muscle Quality

The results are shown in Table 4. Compared with the PC group, hardness and chewiness of muscle in the HL group were significantly decreased, while the crude lipid content and gumminess were observably augmented (*P* < 0.05). Significant increase in the crude lipid content, hardness, and chewiness of muscle was detected in the HS group compared with the PC group (*P* < 0.05). Significant reduction in the gumminess of muscle was found in the HS group compared with the PC group (*P* < 0.05). There were not differences in the crude protein, ash, moisture, elasticity, and adhesiveness of muscle among all groups.

### 3.3. Plasma Biochemistry Measurements

The outcomes are shown in Table 5. TC content in the HL and HS groups significantly increased compared to the PC group (*P* < 0.05). In the HL group, TG, GLU, ALT, and AST contents of plasma evidently raised (*P* < 0.05) and significantly reduced in the HS group. Compared to the HL group, the GR level in the HS group significantly increased (*P* < 0.05). No obvious changes were detected in TP level of plasma among all groups (*P* > 0.05).

### 3.4. Hematological Parameters Measurements

**Figure 1:**
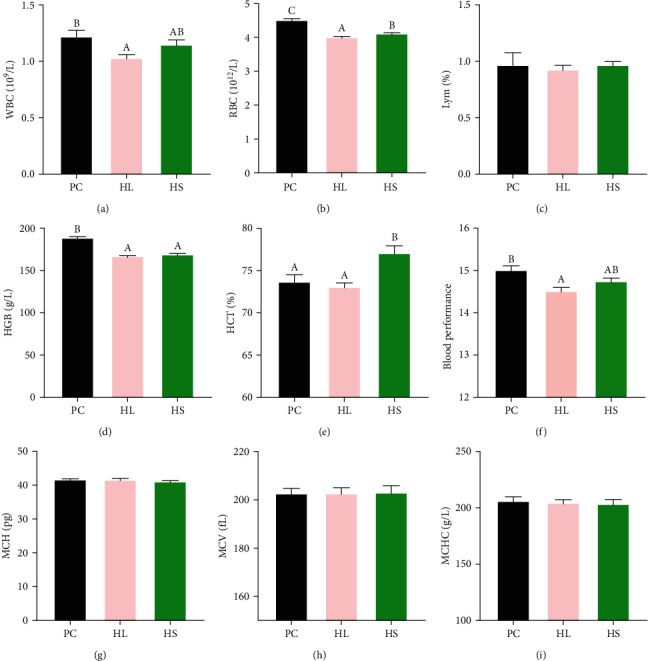
Hematological parameters in *T. ovatus* fed experimental diets: (a) WBC content, (b) RBC content, (c) Lym content, (d) HGB content, (e) HCT content, (f) blood performance content, (g) MCH content, (h) MCV content, and (i) MCHC content. Values are means ± SEM of three replications. Different letters above the bar indicate statistical differences (*P* < 0.05).

The results showed the fish in the HL group significantly reduced the WBC and RBC contents of blood in comparison to those in the PC group (Figure 1, *P* < 0.05). Both in the HL group and the HS group, fish had a significantly lower HGB contents than those in the PC group (Figure 1, *P* < 0.05). In comparison to the PC group, HCT content significantly raised in the HS group (Figure 1, *P* < 0.05). The BP in the HL group had a significant reduction compared to the PC group (Figure 1, *P* < 0.05). However, no significant alteration was detected in Lym, MCV, MCH, and MCHC concentrations within the blood of golden pompano among the three groups (Figure 1, *P* > 0.05).

### 3.5. Hepatic Morphology and Lipid Metabolism Parameters Measurements

**Figure 2:**
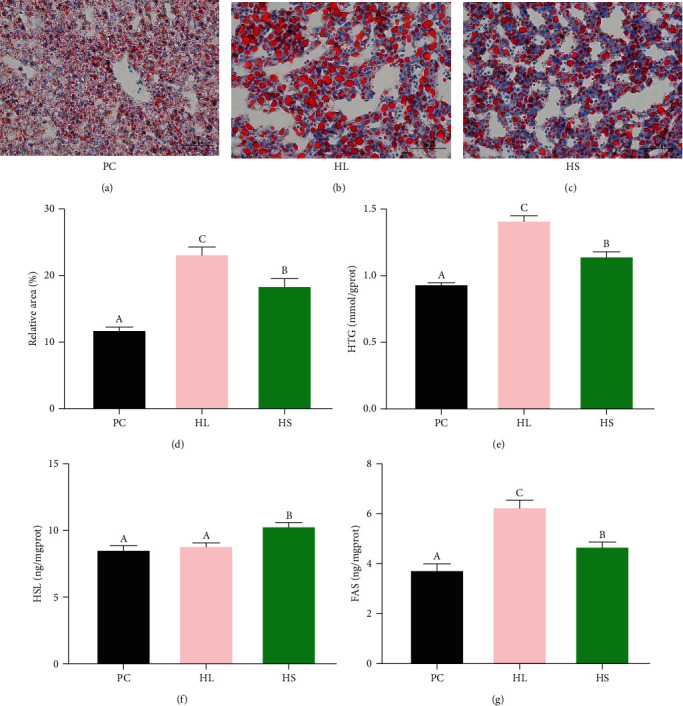
The oil red O sections of the liver (400×) and lipid metabolism parameters in *T. ovatus* fed experimental diets: (a) PC group, (b) HL group, (c) HS group, (d) the relative area of red lipid droplets in the hepatic section area (%), (e) hepatic TG content, (f) HSL content, and (g) FAS content. Values are means ± SEM of three replications. Different letters above the bar indicate statistical differences (*P* < 0.05).

The result of oil red O staining showed significantly larger volume and content of lipid droplets in the HL group than that in the PC group (Figures 2(a), 2(b), 2(c), and 2(d)) and supplementation with sodium acetate in the HL diet evidently decreased the volume and content of lipid droplets (*P* < 0.05). The results of lipid metabolism indicators are shown in Figures 2(e), 2(f), and 2(g). The golden pompano fed HL diet augmented significantly HTG content in comparison to the fish fed PC diet, while sodium acetate supplementation in HL diet decreased HTG levels (*P* < 0.05). Compared to the PC group, the hepatic HSL activity was not obvious alteration in the HL group, whereas the HSL activity significantly raised in the HS group (*P* < 0.05). A significantly higher FAS activity was found in the HL group than that in the PC and HS groups (*P* < 0.05).

### 3.6. Intestinal Morphology

**Figure 3:**
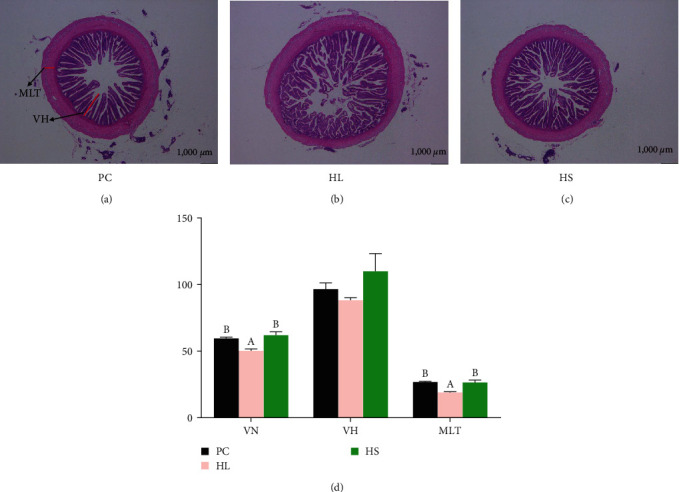
Intestinal histology (20x) of *T. ovatus* fed experimental diets: (a) PC group, (b) HL group, (c) HS group, and (d) morphological parameters of intestine including villus number (VN), villus height (VH), and muscular layer thickness (MLT). Values are means ± SEM of three replications. Different letters above the bar indicate statistical differences (*P* < 0.05).

The outcomes are presented in Figure 3, HL diet caused notable changes to intestinal morphology of juvenile golden pompano. Compared to the PC group, VN and MLT in the HL group significantly reduced, whereas sodium acetate supplementation in the HL group obviously augmented these parameters (*P* < 0.05). There were no obvious changes in VH among 3 groups (*P* > 0.05).

### 3.7. Intestinal Microbiota Analysis

**Figure 4:**
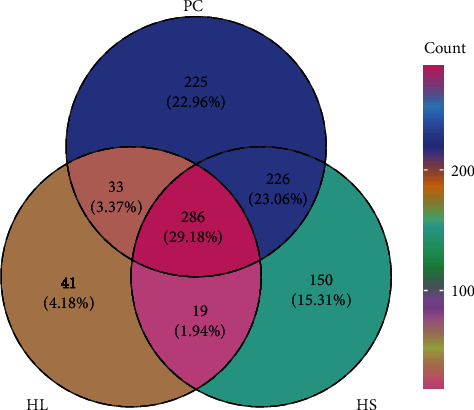
Venn analysis at OTU level of intestinal microbiota in *T. ovatus* fed experimental diets.

We performed a 16S rRNA gene sequencing of the microbiota. Gut microbial alpha diversity is shown in Table 6. The ACE, Chao1, Shannon, and Simpson indices were not obvious difference among these groups (*P* > 0.05). The venn diagram analysed the similarity and difference of OTU among the groups (Figure 4). There were 286 common OTUs in the PC, HL, and HS groups. The additional 33, 19, and 226 OTUs were shared in the PC and HL groups, the HL and HS groups as well as the PC and HL groups, respectively. In addition, 225, 41, and 150 OTUs were uniquely identified in the PC, HL, and HS groups, respectively.

The primary microbiota composition at the phylum level of *T. ovatus* is illustrated in Table 7. The overcomes indicated that Proteobacteria was the most primary phylum, which had the highest proportion in the 3 groups. Compared to the PC group, relative abundance of Tenericutes significantly decreased, while Firmicutes and Fusobacteria significantly increased in the HS group (*P* < 0.05). Compared to the HL group, HS group significantly decreased relative abundance of Proteobacteria and Tenericutes, and significantly increased proportion of Firmicutes, Bacteroidetes, Fusobacteria, and Spirochaetae (*P* < 0.05). The proportion of Cyanobacteria and Actinobacteria showed nonsignificant changes among the 3 groups. Besides, the research concentrated on the top 10 genus with the highest abundance of *T. ovatus* (Table 8). In the HS group, relative abundance of *Mycoplasma* and *Vibrio* was significantly lower compared to the PC and HL groups (*P* < 0.05), while *Bacteroidales_S24-7_group_norank*, *Lachnospiraceae_NK4A136_group*, *Cetobacterium*, and *Bacteroides* were significantly higher than that in the PC and HL groups (*P* < 0.05). Compared to PC group, the HL and HS groups significantly decreased the relative abundance of *Ruegeria* (*P* < 0.05). However, there was no significant difference in the proportion of *Photobacterium*, *Synechococcus*, and *Alloprevotella* among 3 groups.

**Figure 5:**
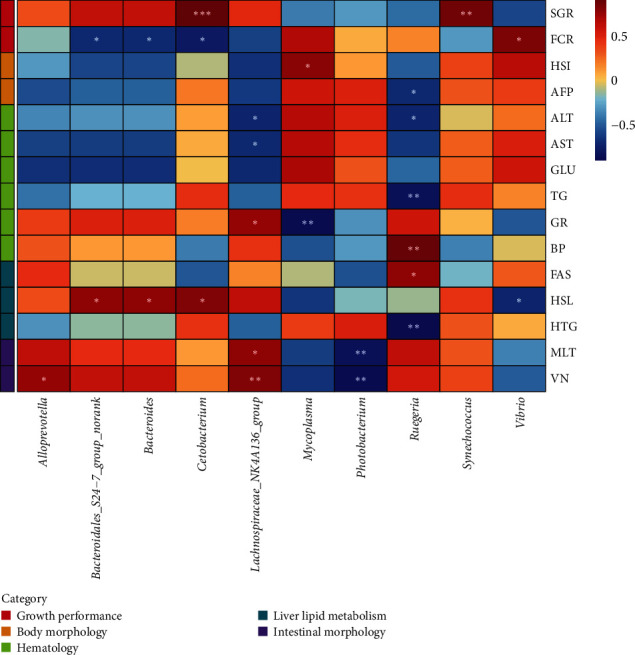
Heatmap of the Spearman's correlation analysis of gut microbiota (*Mycoplasma*, *Photobacterium*, *Bacteroidales_S24-7_group_norank*, *Vibrio*, *Synechococcus*, *Ruegeria*, *Lachnospiraceae_ NK4A136_group*, *Cetobacterium*, *Bacteroides*, *Alloprevotella*) and physiological indexes (SGR, FCR, HSI, AFP, GLU, TG, ALT, AST, GR, BP, VN, MLT, HSL, FAS, HTG) of juvenile *T. ovatus*.  ^*∗*^*P* < 0.05,  ^*∗∗*^*P* < 0.01,  ^*∗∗∗*^*P* < 0.001.

**Figure 6:**
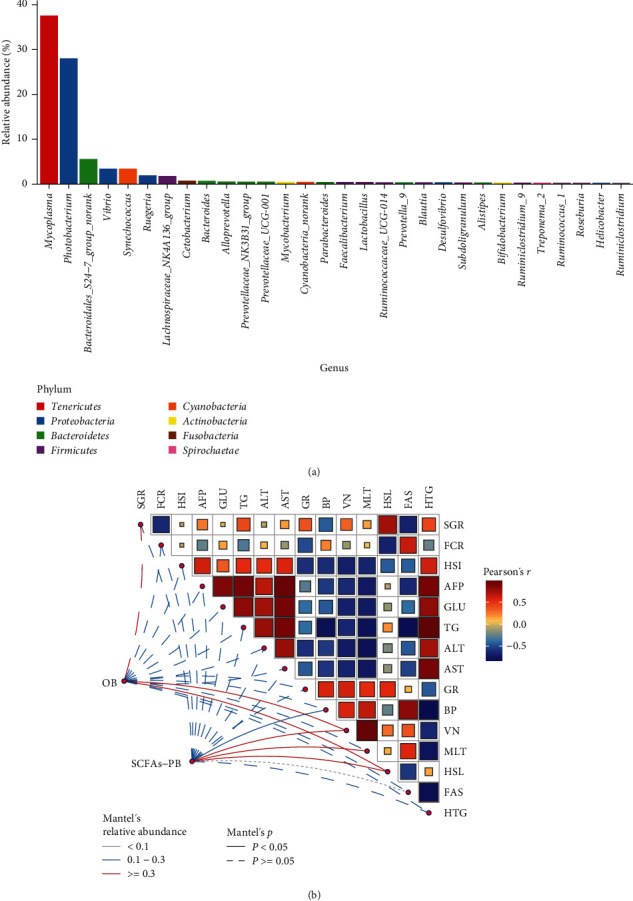
(a) Relative abundance of top 30 genus of intestinal microbiota in *T. ovatus* fed experimental diets. (b) Mantel test of top 30 genus in abundance and physiological biomarkers (SCFAs-PB, SCFAs-producing bacteria, and OB). The dimensions of the square within the matrix represent the relativity (a red square signifies a positive correlation, while a blue square denotes a negative correlation. The deeper the color, the stronger the correlation). The lines outside the matrix reveals the correlation between the genus and physiological indicators (solid lines signify significant correlations, and dashed lines represent nonsignificant correlations).

Spearman's correlation analysis was conducted to investigate the relationship between the top 10 genera of gut microbiota and physiological biomarkers in *T. ovatus* (Figure 5). *Bacteroidales_S24-7_group_norank*, *Bacteroides*, and *Cetobacterium* demonstrated a positive correlation with HSL, but a negative correlation with FCR (*P* < 0.05). On the other hand, *Vibrio* showed a negative correlation with HSL while a positive correlation with FCR (*P* < 0.05). *Lachnospiraceae_NK4A136_group* was positively correlated with VN, MLT, and GR while negatively correlated with ALT and AST (*P* < 0.05). *Mycoplasma* exhibited a negative correlation with GR while a positive correlation with HSI (*P* < 0.05). *Ruegeria* positively related with BP and FAS while negatively related with HTG, TG, ALT, and AFP (*P* < 0.05). Finally, strong positive correlation was found in *Cetobacterium* and SGR, *Synechococcus* and SGR, *Alloprevotella* and VN, while negative correlation in *Photobacterium* and intestinal morphology (MLT, VN; *P* < 0.05). Furthermore, we counted the top 30 abundant genera and found that 22 genera belong to SCFAs-producing bacteria (SCFAs-PB) and only 8 strains belong to other bacteria (OB) (Figure 6(a); Table S1). The relationship between the top 30 abundant genera and physiological indices were examined by the Mantel test (Figure 6(b)). The results showed that SCFAs-PB had strong correlation with BP, VN, MLT, and HSL, while OB had a significantly positive connection with VN and HSL (*P* < 0.05).

## 4. Discussion

Sodium acetate is regarded as a cost-effective feed additive, which can promote growth, enhance immunity, and improve inflammation of fish [24, 29, 30]. However, the information is still limited whether sodium acetate can relieve the adverse status caused by HL diets. In this study, dietary sodium acetate supplementation in HL diets promoted the growth and reduced the FCR of *T. ovatus* compared to the HL diets, which was similar with the outcomes of large-scale shovel-jaw fish [14] and Nile tilapia [13]. Good growth performance is known to be correlated with the healthy intestinal histology. Earlier research indicated intestine contributed to the digestion and absorption of nutrients, which was strongly associated with growth [31]. In the current study, the villus number and muscular layer thickness of intestine evidently reduced in the HL group, and these parameters obviously recovered when sodium acetate was supplemented in HL diets. Increased villus number and muscular layer thickness can enlarge the absorption area for nutrients in aquatic beings [32, 33]. Therefore, these findings indicated sodium acetate supplementation could improve the damaged intestinal histology parameters induced by HL diets thus promote the growth of golden pompano.

Our results found higher HSI and AFP were presented in golden pompano fed HL diets, and these parameters were obviously decreased when fish fed HS diets. Similar phenomena were found in the study of Nile tilapia [13]. The HSI and liver histology can further reflect the amount of hepatic lipid deposition [6]. The hepatic oil red O section in this study displayed sodium acetate addition alleviated the increased area of lipid droplet caused by HL diets in golden pompano. As expected, the change of the hepatic TG content in the current study presented the same trend as the change of lipid deposition. Earlier research in blunt snout bream reported hepatocyte treated with 5 mM sodium acetate for 24 hr relieved lipid accumulation [12]. Prior research demonstrated sodium acetate addition quieted the incremental TG of liver in large-scale shovel-jaw fish fed HL diet [14]. Similarly, dietary sodium acetate addition reduced hepatic lipid deposition induced by HL diets in Nile tilapia [13]. These phenomena were consistent with our study. FAS and HSL as key metabolic enzyme are, respectively, involved in lipogenesis and lipolysis, which play critical roles in effectively regulating abnormal lipid deposition [34]. Further detection for the activity of lipid metabolism enzymes in this research revealed sodium acetate supplementation could meliorate hepatic lipid metabolism by enhancing the HSL activity and inhibiting the FAS activity. Studies pointed out that dietary sodium acetate decreased hepatic lipid accumulation of fish fed HL diets by upregulating the expression of lipolytic genes [13, 14], which all supported our results. Interestingly, in this study, the addition of sodium acetate did not significantly decrease the lipid deposition of muscle in golden pompano, which indicated sodium acetate may specifically or preferentially target the liver tissue to regulate lipid metabolism rather than muscle.

Plasma biochemistry is regarded as a reliable health index mirroring nutrition, stress, and the overall well-being of fish [35]. In the current study, sodium acetate addition obviously alleviated the increased TG content caused by HL diets, and also reduced the high TC level caused by HL diets though it is not significant. These results were supported by recent studies on Nile tilapia and large-scale shovel-jaw fish [13, 14]. Moreover, our results displayed sodium acetate supplementation reduced the high GLU level caused by HL diets, which aligned with the outcomes observed in Nile tilapia [13]. Taken together, sodium acetate had hypolipidemic and hypoglycemic effects for fish fed HL diets. In aquaculture research, AST and ALT are generally assessed to gauged hepatic physiological state [36]. Elevated levels of ALT and AST are recognized as indicators of liver damage in fish [37]. Previous studies demonstrated dietary sodium acetate could reduce ALT and AST level of plasma [14, 24, 30]. In this study, the content of AST and ALT evidently raised in the HL group, and these parameters obviously declined in the HS group, suggesting that sodium acetate could ameliorate HL-induced liver oxidative damage.

Hematological indices serve as crucial indicators to evaluate nutrition value of fish diets and monitoring the well-being of fish [38, 39]. Studies revealed dietary lipid level could affect hematological factors of fish [40, 41]. In this study, the hematological parameters including WBC, RBC, and HGB level of golden pompano fed HL diets significantly reduced. Meanwhile, sodium acetate supplementation in HL diets increased these parameters. In fish, RBC, WBC, and HGB are commonly employed as nutritional indicators, and they are also engaged in regulation of immune function within the organism [42, 43]. These results suggested HL diets caused metabolic and immune disturbance in blood, while the addition of sodium acetate could improve these phenomena. However, hematological indices occasionally do not exhibit a uniform pattern, rendering them unreliable as individual health markers. BP as a new formula has been confirmed more trustworthy and precise for assessing fish well-being and growth because it effectively avoids the limitations of evaluating experimental effectiveness based on a single indicator [27, 44, 45]. Generally, higher blood performance values indicate better health state. In this study, the addition of sodium acetate to the HL diets can improve the decline in the blood performance caused by high lipid, indicating that sodium acetate could enhanced the immunity and health status of golden pompano fed HL diets.

The gut microflora participates in the nutritional metabolism and metabolic well-being of fish [46]. The current data have indicated gut microflora of fish is dynamic and could be manipulated via SCFAs that contribute to altering microbial diversity by boosting the colonization of probiotics [24, 47, 48, 49]. In the current study, the alpha diversity of gut microbiota showed increased trends in golden pompano fed HL diets supplemented with sodium acetate. In any event, an increased microbial abundance ought to be regarded as a favorable outcome, as it could potentially offer additional metabolic abilities to the host, thereby enhancing its health condition [50]. Venn diagrams in this study showed that common OTUs between the PC and HS were higher than those between the PC and HL group, which indicated the structure of microbiome in HS group was more similar with the PC group. Consistent with our study, previous studies revealed the dominant phyla of golden pompano were Proteobacteria, Tenericutes, Bacteroidetes, and Firmicutes, while the dominant genera presented the diversity [51, 52, 53], indicating that the genera of intestinal microbiota were more susceptible to the external environment than the phyla.

Next, we examined the constitution of gut microbiota. Our results found that the inclusion of sodium acetate in HL diets significantly reduced the abundance of Proteobacteria and Tenericutes. The high abundance of Proteobacteria and Tenericutes could be linked to inflammation and metabolic syndrome, which could cause an imbalance in the intestine microbiota [52, 54, 55, 56]. Consistently, *Vibrio* and *Mycoplasma*, as primarily genus, respectively, represented Proteobacteria and Tenericutes, also significantly decreased in the HS group. *Vibrio* and *Mycoplasma* were deemed to potential pathogens and closely associated with host health such as growth, lipid metabolism, and immunity [51, 57, 58]. According to Spearman's correlation analysis of this study, *Vibrio* and *Mycoplasma* had a strong correlation with FCR, HSI, GR, and HSL, which revealed the inclusion of sodium acetate could improve the health status of *T. ovatus* by restraining the abundance of *Vibrio* and *Mycoplasma*. In addition, the findings of this study showed that the proportion of Firmicutes, Bacteroidetes, and Fusobacteria of *T. ovatus* were increased following sodium acetate supplementation compared to the fish fed HL diets. *Lachnospiraceae_NK4A136 _group*, belonging to Firmicutes, has been confirmed as a probiotic [59]. A previous study found increased abundance of intestinal *Lachnospiraceae_NK4A136_group* was benefit for improving lipid metabolism in high-fat diet-fed mice [60]. Bacteroidetes phylum was mainly represented by *Bacteroidales_S24-7_group_norank* and *Bacteroides*. Increased abundance of the two genera was correlated with decreased contents of the inflammatory molecule and cholesterol [61, 62]. *Cetobacterium*, belonging to Fusobacteria, is rich in vitamin B_12_ and can promote the health of fish. A research on zebrafish revealed intestinal *Cetobacterium* regulated glucose metabolism and highlighted an effect of acetate in mediating the mechanism [63]. Previous study has also confirmed that exogenous addition of *Cetobacterium* could decrease the levels of serous AST and ALT, and lipid deposition in liver [64]. In the present study, the relative abundance of these 4 genera (*Lachnospiraceae_NK4A136_group*, *Bacteroidales_S24-7_group_norank*, *Bacteroides*, and *Cetobacterium*) had significant increment in the HS group. Moreover, *Bacteroidales_S24-7_group_norank*, *Bacteroides*, and *Cetobacterium* showed strongly positive correlation with HSL and inverse relationship with FCR. *Lachnospiraceae_NK4A136_group* displayed significant negative correlation with AST and ALT, and positive relationship with MLT and VN. These findings clarified improvement of physiological metabolism of *T. ovatus* fed HS diets was regulated by the incremental relative abundance of these genera.

Although some strains such as *Prevotellaceae_UCG-001*, *Faecalibacterium*, and *Lactobacillus* possessed low proportion in the intestine, these nondominant bacteria have crucial actions [65]. Therefore, we analysed the relative abundance of top 30 abundant genera and explored the relationship between these genera and physiological indices by the Mantel test. Our study displayed that SCFAs-PB significantly affected BP, VN, MLT, and HSL of *T. ovatus*. Previous studies pointed out SCFAs produced by SCFAs-PB were mainly used as the energy source for intestinal epithelial cells, while a limited portion of SCFAs reaches the blood and liver, where they were served as substrates for the tricarboxylic acid cycle and gluconeogenesis [9, 66, 67]. Earlier research found the changes of the gut microbiota were associated with the hematological indicators [68]. These findings indicated the production of SCFA from SCFA-PB may be an important reason for the variable of the physiological and biochemical indicators of *T. ovatus*. In short, sodium acetate could alter the gut microbiota to influence growth and physiological status of golden pompano, which may be the key reason for its ability to mitigate the negative effects of HL diet.

## 5. Conclusion

In the present study, inclusions of sodium acetate in HL diets alleviated the abnormal physiological indices such as poor growth, high blood sugar and lipid, excessive hepatic lipid deposition, and intestine injury of golden pompano. Additionally, dietary sodium acetate addition improved the intestinal microbiota via reducing the pathogenic bacteria (*Mycoplasma* and *Vibrio*) and enriching the probiotics (*Lachnospiraceae_NK4A136_group*, *Bacteroides*, *Cetobacterium*, and *Bacteroidales_S24-7_group_norank*). Our study revealed that these probiotics could play key roles in ameliorating adverse symptoms induced by HL diets.

## Figures and Tables

**Table 1:** Formulation and proximate analysis of the experimental diets (dry weight).

Ingredients	PC	HL	HS
Fish meal	25.00	25.00	25.00
Soybean meal	15.00	15.00	15.00
Soy protein concentrate	10.00	10.00	10.00
Peanut meal	10.00	10.00	10.00
Pork powder	6.00	6.00	6.00
Brewers dried yeast	5.00	5.00	5.00
Wheat flour	19.70	13.56	13.56
Fish oil	6.00	12.00	12.00
Soybean lecithin	1.00	1.00	1.00
Vitamin and mineral mix^a^	1.00	1.00	1.00
Choline chloride	0.50	0.50	0.50
Monocalcium phosphate	0.50	0.50	0.50
L-methionine	0.20	0.20	0.20
Antioxidant	0.10	0.10	0.10
Zeolite powder	—	0.14	—
Sodium acetate^b^	—	—	0.14
Proximate composition (%)			
Moisture	7.59	7.00	7.17
Crude protein	43.82	43.25	43.21
Crude lipid	10.33	16.27	16.38
Ash	12.00	12.13	12.14

^a^Vitamin premix (mg or g kg^−1^ diet): thiamin, 25 mg; riboflavin, 45 mg; pyridoxine HCl, 20 mg; vitamin B_12_, 0.1 mg; vitamin K_3_, 10 mg; inositol, 800 mg; pantothenic acid, 60 mg; niacin acid, 200 mg; folic acid, 20 mg; biotin, 1.20 mg; retinal acetate, 32 mg; cholecalciferol, 5 mg; *α*-tocopherol, 120 mg; ascorbic acid, 2,000 mg; choline chloride, 2,500 mg; ethoxyquin 150 mg; wheat middling, 14.012 g. Mineral premix (mg or g kg^−1^ diet): NaF, 2 mg; KI, 0.8 mg; CoCl_2_ · 6H_2_O (1%), 50 mg; CuSO_4_ · 5H_2_O, 10 mg; FeSO_4_ · H_2_O, 80 mg; ZnSO_4_ · H_2_O, 50 mg; MnSO_4_ · H_2_O, 60 mg; MgSO_4_ · 7H_2_O, 1,200 mg; Ca (H_2_PO_4_)_2_·H_2_O, 3,000 mg; NaCl, 100 mg; zoelite, 15.447 g. ^b^Sodium acetate (purity ≥99%) provided by Sangon Biotech (Shanghai) Co., Ltd.

**Table 2:** Growth performance in *T. ovatus* fed experimental diets.

Parameters	PC	HL	HS
IBW (g)	12.88 ± 0.03	12.89 ± 0.08	12.87 ± 0.04
FBW (g)	122.09 ± 0.25^a^	126.51 ± 1.59^ab^	132.24 ± 2.64^b^
WGR (%)	847.92 ± 3.45^a^	881.30 ± 10.93^ab^	927.76 ± 20.88^b^
SGR (%/d)	4.02 ± 0.01^a^	4.08 ± 0.02^ab^	4.16 ± 0.04^b^
FCR	1.48 ± 0.04^b^	1.43 ± 0.01^ab^	1.37 ± 0.02^a^
Survival rate (%)	98.33 ± 1.67	100.00 ± 0.00	98.33 ± 1.67

Values are means ± SEM of three replications. Means in the same line with different superscripts are significantly different (*P* < 0.05). IBW, initial body weight; FBW, final body weight; WGR, weight gain rate; SGR, specific growth rate; FCR, feed conversion ratio; FI, feed intake.

**Table 3:** Body morphologic indices in *T. ovatus* fed experimental diets.

Parameters	PC	HL	HS
VSI (%)	6.32 ± 0.06	6.45 ± 0.08	6.38 ± 0.11
HSI (%)	0.88 ± 0.02^a^	0.96 ± 0.03^b^	0.88 ± 0.02^a^
CF (g/cm^3^)	3.52 ± 0.04^a^	3.54 ± 0.08^a^	3.78 ± 0.00^b^
AFP (%)	0.83 ± 0.09^a^	1.15 ± 0.04^b^	0.94 ± 0.03^a^

Values are means ± SEM of three replications. Means in the same line with different superscripts are significantly different (*P* < 0.05). VSI, viserosomatic index; HSI, hepatosomatic index; CF, condition factor; AFP, abdominal fat percentage.

**Table 4:** Proximate composition and texture characteristics of muscle in *T.ovatus* fed experimental diets.

Parameters	PC	HL	HS
Proximate composition (%)			
Crude protein	17.75 ± 0.11	18.38 ± 0.44	18.61 ± 0.72
Crude lipid	7.46 ± 0.37^a^	9.19 ± 0.13^b^	9.54 ± 0.36^b^
Crude ash	1.74 ± 0.09	1.70 ± 0.05	1.62 ± 0.03
Moisture	71.78 ± 0.37	70.53 ± 0.62	69.78 ± 1.06
Texture characteristics			
Hardness (g)	396.00 ± 2.25^b^	355.83 ± 1.59^a^	404.83 ± 2.85^c^
Elasticity (mm)	1.84 ± 0.04	1.85 ± 0.02	1.77 ± 0.01
Chewiness (mJ)	2.49 ± 0.08^b^	2.18 ± 0.05^a^	2.52 ± 0.10^b^
Adhesiveness (mJ)	0.25 ± 0.01	0.27 ± 0.01	0.27 ± 0.01
Gumminess (g)	95.27 ± 0.39^b^	104.47 ± 0.22^c^	89.27 ± 1.69^a^

Values are means ± SEM of three replications. Means in the same line with different superscripts are significantly different (*P* < 0.05).

**Table 5:** Plasma biochemistry indices in *T. ovatus* fed experimental diets.

Parameters	PC	HL	HS
TC (mmol/L)	5.94 ± 0.42^a^	7.81 ± 0.17^b^	7.31 ± 0.24^b^
TP (g/L)	43.43 ± 3.47	40.10 ± 1.85	41.30 ± 0.95
TG (mmol/L)	1.14 ± 0.03^a^	1.82 ± 0.08^c^	1.48 ± 0.02^b^
GLU (mmol/L)	4.34 ± 0.57^a^	6.49 ± 0.70^b^	4.42 ± 0.31^a^
ALT (U/L)	4.00 ± 0.58^a^	10.50 ± 2.02^b^	4.00 ± 0.58^a^
AST (U/L)	41.67 ± 4.48^a^	63.50 ± 4.91^b^	45.67 ± 0.88^a^
GR (U/L)	3.67 ± 0.20^ab^	2.75 ± 0.20^a^	4.40 ± 0.52^b^

Values are means ± SEM of three replications. Means in the same line with different superscripts are significantly different (*P* < 0.05). TC, total cholesterol; TP, total protein; TG, triglyceride; GLU, glucose; ALT, alanine aminotransferase; AST, aspartate aminotransferase; GR, glutathione reductase.

**Table 6:** Alpha diversity of intestinal microbiota in *T. ovatus* fed experimental diets.

Parameters	PC	HL	HS
ACE	476.00 ± 142.13	546.67 ± 80.70	811.33 ± 117.82
Chao1	484.33 ± 134.72	542.67 ± 78.67	801.67 ± 116.16
Shannon	2.88 ± 0.51	2.34 ± 0.22	3.62 ± 0.56
Simpson	0.17 ± 0.04	0.21 ± 0.02	0.11 ± 0.04

Values are means ± SEM of three replications. Means in the same line with different superscripts are significantly different (*P* < 0.05).

**Table 7:** The relative abundance of identified bacteria on phylum level in the intestine of *T. ovatus* fed experimental diets.

Phylum	PC	HL	HS
Proteobacteria	40.18 ± 3.95^ab^	46.50 ± 4.33^b^	30.53 ± 4.36^a^
Tenericutes	37.53 ± 2.21^b^	41.03 ± 1.57^b^	26.50 ± 2.48^a^
Firmicutes	6.93 ± 1.04^a^	2.45 ± 0.44^a^	16.20 ± 2.35^b^
Bacteroidetes	7.17 ± 4.43^ab^	2.85 ± 1.78^a^	15.52 ± 2.37^b^
Cyanobacteria	4.64 ± 0.91	4.01 ± 0.79	6.30 ± 1.60
Actinobacteria	1.29 ± 0.21	1.69 ± 0.23	1.56 ± 0.27
Fusobacteria	0.15 ± 0.01^a^	0.27 ± 0.09^a^	1.97 ± 0.36^b^
Spirochaetae	0.73 ± 0.14^b^	0.28 ± 0.04^a^	0.93 ± 0.08^b^
Others	1.38 ± 0.21^b^	0.92 ± 0.10^ab^	0.49 ± 0.12^a^

Values are means ± SEM of three replications. Means in the same line with different superscripts are significantly different (*P* < 0.05).

**Table 8:** The relative percentage presence of the top 10 genus in the intestine of *T. ovatus* fed experimental diets.

Genus	PC	HL	HS
*Mycoplasma*	37.45 ± 1.26^b^	41.00 ± 0.30^b^	24.99 ± 2.48^a^
*Photobacterium*	28.23 ± 4.22	36.53 ± 1.89	23.67 ± 7.12
*Bacteroidales_S24-7_group_norank*	3.84 ± 1.62^a^	1.51 ± 0.02^a^	9.46 ± 0.45^b^
*Vibrio*	4.70 ± 0.23^b^	4.68 ± 0.50^b^	1.77 ± 0.39^a^
*Synechococcus*	2.31 ± 0.16	3.26 ± 0.61	5.37 ± 1.38
*Ruegeria*	3.62 ± 0.56^b^	1.13 ± 0.04^a^	1.73 ± 0.07^a^
*Lachnospiraceae_NK4A136_group*	1.23 ± 0.14^a^	0.56 ± 0.06^a^	4.08 ± 0.92^b^
*Cetobacterium*	0.07 ± 0.00^a^	0.26 ± 0.10^a^	1.68 ± 0.67^b^
*Bacteroides*	0.46 ± 0.21^a^	0.20 ± 0.06^a^	1.14 ± 0.18^b^
*Alloprevotella*	0.50 ± 0.29	0.18 ± 0.13	0.92 ± 0.44

Values are means ± SEM of three replications. Means in the same line with different superscripts are significantly different (*P* < 0.05).

## Data Availability

Data were available from the corresponding authors by reasonable request.

## Supplementary Materials

Supplementary Materials Table S1: the numbers of SCFAs-PB of TOP 30 genera.
