# Supplementary material for: Effects of Sodium Acetate Supplementation on Growth, Hematologic and Plasma Biochemical Parameter, Lipid Deposition, and Intestinal Health of Juvenile Golden Pompano Trachinotus ovatus Fed High-Lipid Diets
Source: Aquac Nutr. 2024 Sep 7;2024:7904141. doi: 10.1155/2024/7904141 (PMC11401687; doi:10.1155/2024/7904141)
Supplement: Supplementary Materials — Table S1: the numbers of SCFAs-PB of TOP 30 genera. [file 7904141.f1.docx]

**Table S1** The numbers of SCFAs-PB of TOP 30 genera

| Genus | SCFAs-PB or OB | References |
| --- | --- | --- |
| *Mycoplasma* | OB |  |
| *Photobacterium* | OB |  |
| *Bacteroidales_S24-7_group_norank* | SCFAs-PB | Yang et al., 2020 |
| *Vibrio* | OB |  |
| *Synechococcus* | OB |  |
| *Ruegeria* | SCFAs-PB | Tamanai-Schacoori et al., 2017 |
| *Lachnospiraceae_NK4A136_group* | SCFAs-PB | Yan et al., 2023 |
| *Cetobacterium* | SCFAs-PB | Wang et al., 2021 |
| *Bacteroides* | SCFAs-PB | Su et al., 2023 |
| *Alloprevotella* | SCFAs-PB | Xie et al., 2023 |
| *Prevotellaceae_NK3B31_group* | SCFAs-PB | Huang et al., 2021 |
| *Prevotellaceae_UCG-001* | SCFAs-PB | Zhu et al., 2019 |
| *Mycobacterium* | OB |  |
| *Cyanobacteria_norank* | OB |  |
| *Parabacteroides* | SCFAs-PB | Zhang et al., 2021 |
| *Faecalibacterium* | SCFAs-PB | Ferreira-Halder et al., 2017 |
| *Lactobacillus* | SCFAs-PB | Chang et al., 2021 |
| *Ruminococcaceae_UCG-014* | SCFAs-PB | Huang et al., 2021 |
| *Prevotella_9* | SCFAs-PB | Mosterd et al., 2021 |
| *Blautia* | SCFAs-PB | Ozato et al., 2019 |
| *Desulfovibrio* | SCFAs-PB | Hong et al., 2021 |
| *Subdoligranulum* | SCFAs-PB | Hul et al., 2020 |
| *Alistipes* | SCFAs-PB | Singh et al., 2023 |
| *Bifidobacterium* | SCFAs-PB | Usta-Gorgun et al., 2020 |
| *Ruminiclostridium_9* | SCFAs-PB | Hsiao et al., 2021 |
| *Treponema_2* | OB |  |
| *Ruminococcus_1* | SCFAs-PB | Takahashi et al., 2016 |
| *Roseburia* | SCFAs-PB | Kasahara et al., 2018 |
| *Helicobacter* | OB |  |
| *Ruminiclostridium* | SCFAs-PB | Hu et al., 2022 |

OB, other bacteria; SCFAs-PB, SCFAs-Producing bacteria

Yang C., Xiao Y., Wu Q., et al., 2017. Preventive effect of proanthocyanidin on gut microbiome in dyslipidemic mice. Food Science, 41, 120-126. <https://doi.org/10.7506/spkx1002-6630-20190703-053>

Tamanai-Schacoori Z., Smida I., Bousarghin L., et al., 2017. *Roseburia* spp.: a marker of health? Future Microbiology, 12, 157-170. <https://doi.org/10.2217/fmb-2016-0130>

Yan C., Huang S., Ding H., et al., 2023. Adverse effect of oxidized cholesterol exposure on colitis is mediated by modulation of gut microbiota. Journal of Hazardous Materials, 459, 132057. <https://doi.org/10.1016/j.jhazmat.2023.132057>

Wang A., Zhang Z., Ding Q., et al., 2021. Intestinal *Cetobacterium* and acetate modify glucose homeostasis via parasympathetic activation in zebrafish. Gut Microbes, 13, e1900996. <https://doi.org/10.1080/19490976.2021.1900996>

Su M., Hu R., Tang T., Tang W., Huang C., 2023. Review of the correlation between Chinese medicine and intestinal microbiota on the efficacy of diabetes mellitus. Frontiers in Endocrinology, 13, 1085092. <https://doi.org/10.3389/fendo.2022.1085092>

Zhang S., Wu P., Tian Y., et al., 2021. Gut microbiota serves a predictable outcome of short-term low-carbohydrate diet (LCD) intervention for patients with obesity. Microbiology Spectrum, 9, e00223-21. <https://doi.org/10.1128/Spectrum.00223-21>

Xie J., Tian S., Liu J., et al., 2023. Combination therapy with indigo and indirubin for ulcerative colitis via reinforcing intestinal barrier function. Oxidative Medicine and Cellular Longevity, 2023, 2894695. <https://doi.org/10.1155/2023/2894695>

Huang P., Jiang A., Wang X., et al., 2021. NMN maintains intestinal homeostasis by regulating the gut microbiota. Frontier in Nutrition, 29, 714604. <https://doi.org/10.3389/fnut.2021.714604>

Zhu H., Liang Y., Ma Q., et al., 2019. Xiaoyaosan improves depressive-like behavior in rats with chronic immobilization stress through modulation of the gut microbiota. Biomedicine and Pharmacotherapy, 112, 108621. <https://doi.org/10.1016/j.biopha.2019.108621>

Ferreira-Halder C., Faria A., Andrade S., et al., 2017. Action and function of *Faecalibacterium prausnitzii* in health and disease. Best Practice & Research Clinical Gastroenterology, 31, 643-648. <https://doi.org/10.1016/j.bpg.2017.09.011>

Chang Y., Jeong C., Cheng W., et al., 2021. Quality characteristics of yogurts fermented with short-chain fatty acid-producing probiotics and their effects on mucin production and probiotic adhesion onto human colon epithelial cells. Journal of Dairy Science, 104, 7415-7425. <https://doi.org/10.3168/jds.2020-19820>

Mosterd C., Kanbay M., van den Born H., van Raalte D., Rampanelli E., 2021. Intestinal microbiota and diabetic kidney diseases: the Role of microbiota and derived metabolites inmodulation of renal inflammation and disease progression. Best Practice & Research Clinical Endocrinology & Metabolism, 35, 101484. <https://doi.org/10.1016/j.beem.2021.101484>

Ozata N., Saito S., Yamaguchi T., et al., 2019. *Blautia* genus associated with visceral fat accumulation in adults 20–76 years of age. NPJ Biofilms and Microbiomes, 5, 28. <https://doi.org/10.1038/s41522-019-0101-x>

Hong Y., Sheng L., Zhong L., et al., 2021. *Desulfovibrio* vulgaris, a potent acetic acid-producing bacterium, attenuates nonalcoholic fatty liver disease in mice. Gut Microbes, 13, 1-20. <https://doi.org/10.1080/19490976.2021.1930874>

Hul M., Roy T., Prifti E., et al., 2020. From correlation to causality: the case of *Subdoligranulum*. Gut Microbes, 12, 1849998. <https://doi.org/10.1080/19490976.2020.1849998>

Singh V., Lee G., Son H., et al., 2023. Butyrate producers, “The Sentinel of Gut”: Their intestinal significance with and beyond butyrate, and prospective use as microbial therapeutics. Frontiers in Microbiology, 13, 1103836. <https://doi.org/10.3389/fmicb.2022.1103836>

Usta-Gorgun B., Yilmaz-Ersan L., 2020. Short-chain fatty acids production by Bifidobacterium species in the presence of salep. Electronic Journal of Biotechnology, 47, 29-35. <https://doi.org/10.1016/j.ejbt.2020.06.004>

Hsiao Y., Chen H., Tsai J., et al., 2021. Administration of *Lactobacillus reuteri* combined with *Clostridium butyricum* attenuates cisplatin-induced renal damage by gut microbiota reconstitution, increasing butyric acid production, and suppressing renal inflammation. Nutrients, 13, 2792. <https://doi.org/10.3390/nu13082792>

Takahashi K., Nishida A., Fujimoto T., et al., 2016. Reduced abundance of butyrate-producing bacteria species in the fecal microbial community in Crohn's disease. Digestion, 93, 59-65. <https://doi.org/10.1159/000441768>

Kasahara K., Krautkramer K., Org E., 2018. Interactions between *Roseburia* intestinalis and diet modulate atherogenesis in a murine model. Nature Microbiology, 3, 1461-1471. <https://doi.org/10.1038/s41564-018-0272-x>

Hu W., Huang L., Zhou Z., et al., 2022. Diallyl disulfide (dads) ameliorates intestinal candida albicans infection by modulating the gut microbiota and metabolites and providing intestinal protection in mice. Frontiers in Cellular and Infection Microbiology, 7, 743454. <https://doi.org/10.3389/fcimb.2021.743454>
